# NMR-Based Metabolomic Analysis for the Effects of α-Ketoglutarate Supplementation on C2C12 Myoblasts in Different Energy States

**DOI:** 10.3390/molecules26071841

**Published:** 2021-03-25

**Authors:** Yantong Li, Xiaoyuan Li, Yifeng Gao, Caihua Huang, Donghai Lin

**Affiliations:** 1 Key Laboratory of Chemical Biology of Fujian Province, Department of Chemical Biology, College of Chemistry and Chemical Engineering, Xiamen University, Xiamen 361005, China; yantongli@stu.xmu.edu.cn (Y.L.); xiaoyuan@stu.xmu.edu.cn (X.L.); yifenggao@stu.xmu.edu.cn (Y.G.); 2 Research and Communication Center of Exercise and Health, Xiamen University of Technology, Xiamen 361021, China

**Keywords:** AKG supplementation, biomolecular NMR, metabolomics, myoblasts, metabolic profile

## Abstract

α-Ketoglutarate (AKG) is attracting much attention from researchers owing to its beneficial effects on anti-aging and cancer suppression, and, more recently, in nutritional supplements. Given that glucose is the main source of energy to maintain normal physiological functions of skeletal muscle, the effects of AKG supplementation for improving muscle performance are closely related to the glucose level in skeletal muscle. The differences of AKG-induced effects in skeletal muscle between two states of normal energy and energy deficiency are unclear. Furthermore, AKG-induced metabolic changes in skeletal muscles in different energy states also remain elusive. Here, we assessed the effects of AKG supplementation on mouse C2C12 myoblast cells cultured both in normal medium (Nor cells) and in low-glucose medium (Low cells), which were used to mimic two states of normal energy and energy deficiency, respectively. We further performed NMR-based metabolomic analysis to address AKG-induced metabolic changes in Nor and Low cells. AKG supplementation significantly promoted the proliferation and differentiation of cells in the two energy states through glutamine metabolism, oxidative stress, and energy metabolism. Under normal culture conditions, AKG up-regulated the intracellular glutamine level, changed the cellular energy status, and maintained the antioxidant capacity of cells. Under low-glucose culture condition, AKG served as a metabolic substrate to reduce the glutamine-dependence of cells, remarkably enhanced the antioxidant capacity of cells and significantly elevated the intracellular ATP level, thereby ensuring the normal growth and metabolism of cells in the state of energy deficiency. Our results provide a mechanistic understanding of the effects of AKG supplements on myoblasts in both normal energy and energy deficiency states. This work may be beneficial to the exploitation of AKG applications in clinical treatments and nutritional supplementations.

## 1. Introduction

The skeletal muscle is the largest organ in the human body and maintains normal life activities. Prolonged exercise training or pathological processes of diseases induce muscle damage or insufficient muscle energy supply, at this time, restoration of muscle strength and function is particularly important. Various nutritional supplements have been employed to promote skeletal muscle hypertrophy and enhance sports performance [1]. As the intersection of the organic carbon and nitrogen metabolism and simultaneously a critical intermediate in the TCA cycle, α-Ketoglutarate (AKG) has shown pleiotropic effects for improving muscle performance in clinical and animal experiments [2,3,4,5,6,7,8,9]. For example, AKG can reduce intestinal mucosal damage [8] and inflammation [9], attenuate the development of colorectal cancer [4] and liver fibrosis [5], promote growth [6], and reduce morbidity and delay aging [7]. Previous works have demonstrated that AKG supplementation can alleviate muscle loss by parenteral administration in a trauma model of patients undergoing total hip replacement [2], and promote muscle hypertrophy and protein synthesis through Akt/mTOR signaling pathways [10,11]. In the case of Duchenne muscular dystrophy, AKG supplementation can prevent muscle atrophy and dysfunction through the PHD3/ADRB2-mediated pathway [12]. Our previous work also showed that AKG supplementation can profoundly facilitate the proliferation of C2C12 myoblasts, and alleviate the atrophy of C2C12 myotubes cultured in a no-glucose medium [13]. Given that glucose is the main source of energy to maintain normal physiological functions of skeletal muscle, the effects of AKG supplementation for improving muscle performance are greatly dependent on the glucose level in skeletal muscle. The differences of the AKG-induced effects in skeletal muscle between different energy states are unclear.

AKG participates in the synthesis of amino acids, vitamins, and organic acids and energy metabolism in the body, which involves the conversion of AKG into glutamate by glutamate dehydrogenase, and the subsequent amidation of glutamate with ammonia by glutamine synthetase. AKG provides the energy for cell growth via the TCA cycle and oxidative phosphorylation, and promotes cell metabolism and signaling by interacting with its receptor OXGR (a G protein-coupled receptor) on the cell membrane [14,15]. Moreover, AKG can regulate mitochondrial oxidative metabolism and promote the permissive epigenetic state by mediating early embryonic cell state transition and germ cell development [16]. Furthermore, exercise-induced AKG can stimulate its receptor OXGR1 in the adrenal glands to control thermogenesis and the breakdown of triglycerides in adipose tissue, and produce beneficial effects on metabolisms [17].

However, few studies have been performed to reveal AKG-induced metabolic changes of skeletal muscle in different energy states and the underlying metabolic mechanisms. Recently, metabolomic analysis has been employed to systematically clarify the molecular mechanisms underlying the beneficial effects of nutritional supplementation. Alternations of metabolites acting as the downstream products of gene transcription, may reflect overall metabolic changes intuitively. As particularly suitable techniques for quantitatively detecting alterations of metabolite levels in biofluid, tissues, and cells, high-resolution, ^1^H nuclear magnetic resonance (NMR) spectroscopy has been extensively applied in metabolomic analyses. Significantly, NMR-based metabolomic profiling has several advantages such as high reproducibility, quantitative measurement without prejudice, and convenient sample preparation [18,19]. Previously, we performed NMR-based metabolomic analyses for elucidating both the effects of creatine supplementation on C2C12 myoblasts [20], and the effects of alanyl-glutamine supplementation on myoblasts injured by energy deprivation [21].

In the present work, we conducted NMR-based cellular metabolomic analysis to compare the effects of AKG supplementation on C2C12 myoblasts in two states of normal energy and energy deficiency, which were cultured both in normal medium and in low-glucose medium with or without AKG supplementation. The two different energy states are closely related to different proliferation and differentiation of myoblasts. We addressed the distinctions of AKG-induced intracellular metabolic changes and the underlying metabolic mechanisms between the myoblasts under normal culture condition and those under low-glucose culture condition. Our work may be beneficial to the further exploitation of AKG supplementation in sports and therapeutic fields.

## 2. Results

### 2.1. Proliferation and Differentiation of C2C12 Myoblasts with AKG Supplementation

C2C12 myoblast cells cultured in a normal growth medium with and without AKG supplementation were grouped as Nor-A and Nor, whereas those in low-glucose growth medium with or without AKG supplementation were grouped as Low-A and Low. Consistent with the previous study [10], Nor-A cells with AKG supplementation at a concentration of 2 mm exhibited a significantly enhanced cell viability relative to Nor cells (Figure S1). Therefore, the AKG concentration of 2 mm was used in the experiments for assessing AKG-induced changes in the proliferation and differentiation of myoblasts.

**Figure 1:**
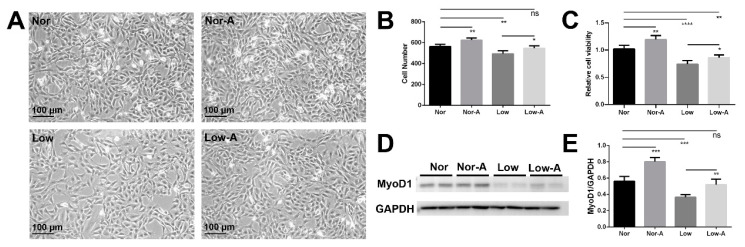
Proliferation and differentiation abilities of C2C12 myoblasts under the conditions of normal culture and low-glucose culture. (**A**) Myoblasts morphologies. (**B**) Cell numbers corresponding to panel A (*n* = 4). (**C**) Cell viabilities relative to Nor cells analyzed by MTS cell proliferation assay (*n* = 5). (**D**) MyoD1 expressions in myoblasts analyzed by western blot. The anti-GAPDH antibody was used to standardize the amount of protein in each lane. (**E**) Statistical analyses corresponding to the panels (**D**) (*n* = 4). * *p* < 0.05, ** *p* < 0.01, *** *p* < 0.001, **** *p* < 0.0001.

Compared with Nor cells, Low cells showed a profoundly declined proliferation rate due to energy deficiency (Figure 1A). Even though AKG supplementation did not cause significantly different morphology of myoblasts in the two energy states, it not only enhanced the proliferation rate of Low cells cultured in a low-glucose medium, but also enhanced that of Nor cells cultured in a normal medium in accordance with previous studies [10,12]. Cell numbers per a given area were counted for the four groups of myoblasts (*n* = 4 for group): Nor, 563.8 ± 10.4; Nor-A, 624.0 ± 10.8; Low, 493.8 ± 14.5; Low-A, 547.0 ± 11.7 (Figure 1B). It is worth noting that Low-A cells did not display statistically different cell numbers from Nor cells, indicating that AKG supplementation could recover cell numbers for the myoblasts under low-glucose culture condition (Figure 1B).

In addition, the MTS assay was performed to quantitatively compare proliferation rates of the four groups of cells (Figure 1C). Low cells had a distinctly decreased proliferation rate compared with Nor cells, indicating that the low-glucose medium disfavored the proliferation of cells. Significantly, AKG supplementation enhanced the proliferation rates of Nor-A and Low-A cells relative to Nor and Low cells, respectively. Note that Low-A cells showed a proliferation ability lower than Nor cells, implying that AKG supplementation only partially restored the proliferation of cells cultured in a low-glucose medium.

The expression of the myogenic differentiation 1 (MyoD1) protein is usually used to characterize the differentiation ability of cells. We, thus, quantitatively compared the expressions of MyoD1 between the four groups of cells (Figure 1D). Low cells showed a profoundly declined differentiation ability compared with Nor cells. Significantly, AKG supplementation up-regulated differentiation abilities of cells cultured both in normal medium and in low-glucose medium, as indicated by about a 30% increase of MyoD1 expression in Nor-A and Low-A cells. Notably, Low-A cells did not display statistically significantly different MyoD1 expression from Nor cells, implying the high efficiency of AKG supplementation for restoring the differentiation ability of cells cultured in a low-glucose medium.

Furthermore, we analyzed myotube differentiation abilities for the four groups of C2C12 myoblasts. Myotubes were formed through the fusion of myoblasts cultured in normal and low-glucose differentiation media with or without AKG supplementation (Figure S3). Morphologies of the C2C12 myotubes showed that the low-glucose culture impaired the myotube differentiation ability of cells, and AKG supplementation could promote myotube differentiation of myoblasts cultured both in normal medium and in a low-glucose medium.

### 2.2. NMR Spectra of Aqueous Extracts of C2C12 Myoblasts

**Figure 2:**
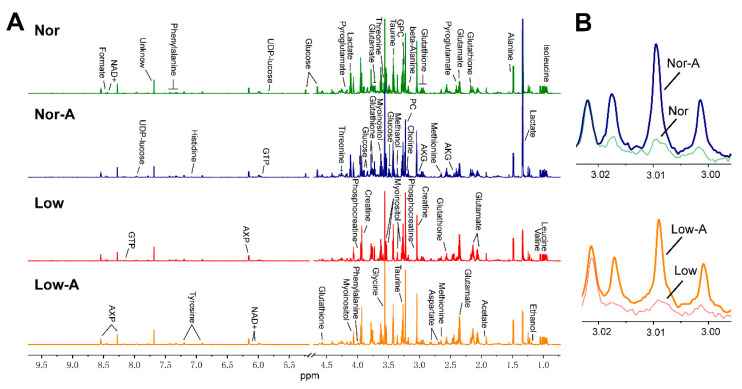
Average 850 MHz ^1^H nuclear magnetic resonance (NMR) spectra recorded on aqueous extracts derived from the Nor, Nor-A, Low, and Low-A groups of C2C12 myoblasts. (**A**) Comparison of the average NMR spectra of the four groups. The vertical scales were kept constant in all the ^1^H NMR spectra. The water region (4.7–5.2 ppm) was removed. (**B**) Local amplified regions of α-Ketoglutarate (AKG) peaks. Blue/green/yellow/red line: spectral regions from the Nor/Nor-A/Low/Low-A groups. AKG, α-ketoglutarate; PC, O-phosphocholine; GPC, sn-glycero-3-phosphocholine; UDP-glucose, Uridine diphosphate glucose; GTP, sn-glycero-3-phosphocholine; NAD+, nicotinamide adenine dinucleotide; AXP, adenine mono/di/tri phosphate.

Typical 850 MHz ^1^H NMR spectra were recorded on aqueous extracts derived from the Nor, Nor-A, Low, and Low-A groups of C2C12 myoblasts (Figure 2A). A total of 34 metabolites were assigned and summarized in Table S1. The resonance assignments of the metabolites were confirmed by using 2D ^1^H-^13^C HSQC and ^1^H-^1^H TOCSY spectra (Figures S4 and S5). The visual inspection of the NMR spectra indicated that culturing myoblasts with AKG supplementation resulted in significant accumulations of intracellular AKG in Nor-A and Low-A cells (Figure 2B).

### 2.3. Multivariate Data Analysis for Exploring Cellular Metabolic Profiles

**Figure 3:**
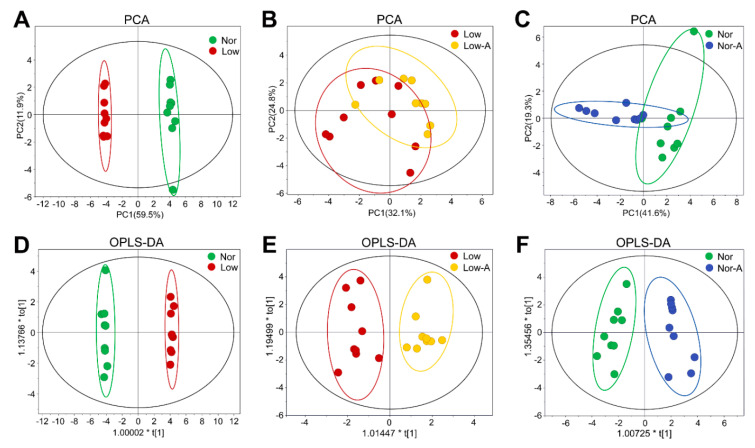
Multivariate analyses for ^1^H NMR spectra recorded on aqueous extracts derived from C2C12 myoblasts of the Nor, Nor-A, Low, and Low-A groups. (**A**–**C**) PCA scores plots of the Low and Nor groups, the Low-A and Low groups, the Nor-A and Nor groups; (**D**–**F**) OPLS-DA scores plots of the Low and Nor groups (R2: 0.999; Q2: 0.996), the Low-A and Low groups (R2: 0.918; Q2: 0.761), the Nor-A and Nor groups (R2: 0.927; Q2: 0.838). The ellipses indicate the 95% confidence limits.

We further performed multivariate data analysis on the NMR spectral data for metabolic profiling of the four groups of C2C12 myoblasts. We firstly established three unsupervised PCA models with the first two components (PC1, PC2) to overview the grouping trends and reveal metabolic differences between the groups of myoblasts. The PCA score plots show that the metabolic profile of cells cultured in a low-glucose medium was distinctly distinguished from that cultured in normal medium (Figure 3A), and AKG supplementation significantly changed the metabolic patterns of the cells cultured both in normal medium and in low-glucose medium (Figure 3B,C). However, the metabolic difference between the Nor-A and Nor groups was larger than that between the Low-A and Low groups, implying that the effects of AKG supplementation on the metabolic profile of myoblasts are greatly dependent on the energy state of cells.

Furthermore, we established three supervised OPLS-DA models to illustrate metabolic separations between the four groups of myoblasts (Figure 3D–F). As expected, the OPLS-DA models maximized the metabolic distinctions between the four groups by reserving the correlated orthogonal variable information and filtering out uncorrelated orthogonal variable information. Furthermore, we performed random permutation tests (*n* = 200) to evaluate the reliabilities of the OPLS-DA models (Figure S6), which indicate the validities of the established OPLS-DA models.

### 2.4. Identifications of Differential and Important Metabolites

**Figure 4:**
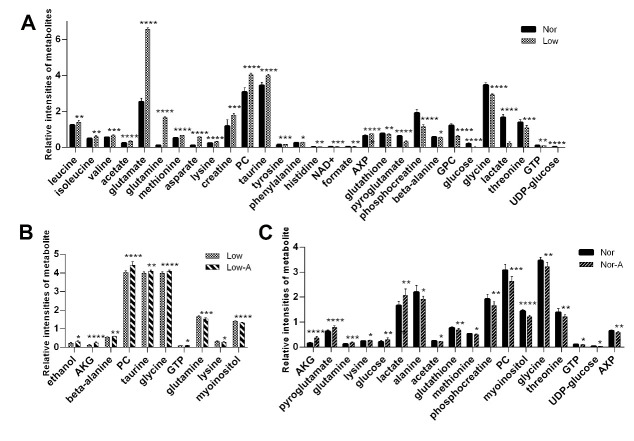
Relative intensities of differential metabolites identified from pairwise comparisons between the four groups of C2C12 myoblasts. (**A**) Low vs. Nor; (**B**) Low-A vs. Low; (**C**) Nor-A vs. Nor. * *p* < 0.05, ** *p* < 0.01, *** *p* < 0.001, **** *p* < 0.0001. *n* = 9 for each group.

To quantitatively compare metabolite levels between the four groups of C2C12 myoblasts, we calculated relative levels of the identified metabolites based on their relative NMR integrals (Table S2). Dramatically, AKG supplementation increased intracellular AKG levels in the Nor-A and Low-A groups, but did not significantly change those in the Nor and Low group. We conducted Student’s *t*-test to identify differential metabolites with a criterion of *p* < 0.05 (Figure 4). The comparison of Nor vs. Low identified 29 differential metabolites (Figure 4A), including 18 enhanced metabolites (leucine, isoleucine, valine, acetate, glutamate, glutamine, methionine, aspartate, lysine, creatine, PC (O-phosphocholine), taurine, tyrosine, phenylalanine, histidine, NAD+, formate, AXP), and 11 declined metabolites (glutathione, pyroglutamate, phosphocreatine, beta-alanine, GPC, glucose, glycine, lactate, threonine, GTP, UDP-glucose). The comparison of Low-A vs. Low identified 10 differential metabolites (Figure 4B), including 7 increased metabolites (ethanol, AKG, beta-alanine, PC, taurine, glycine, and GTP) and 3 decreased metabolites (glutamine, lysine, and myoinositol). The comparison of Nor-A vs. Nor identified 18 differential metabolites (Figure 4C), including 6 up-regulated metabolites (AKG, pyroglutamate, glutamine, lysine, glucose, lactate), and 12 down-regulated metabolites (alanine, acetate, glutathione, methionine, phosphocreatine, PC, myoinositol, glycine, threonine, GTP, UDP-glucose, and AXP).

**Figure 5:**
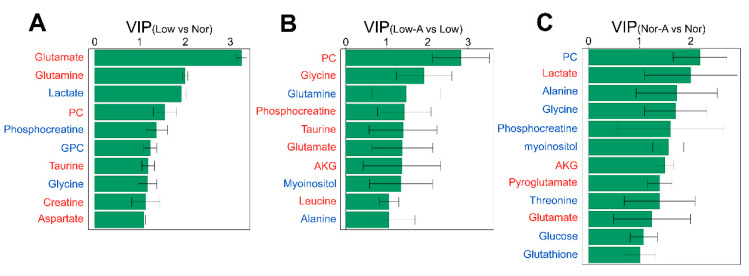
VIP scores of important metabolites identified from pairwise comparisons between the four groups of C2C12 myoblasts. (**A**) Low vs. Nor.; (**B**) Low-A vs. Low; (**C**) Nor-A vs. Nor. Red/Blue font denotes increased/decreased level of the metabolite.

Furthermore, we used the OPLS-DA models to identify important metabolites with a criterion of VIP > 1 (Figure 5). Totally, 12, 10, and 10 important metabolites were identified from the OPLS-DA models of Nor-A vs. Nor, Nor vs. Low, Low-A vs. Low.

The combination of the identified important metabolites and differential metabolites gave characteristic metabolites (Table 1). The pairwise comparisons of Low vs. Nor, Low-A vs. Low, Nor-A vs. Nor identified 10, 6, and 10 characteristic metabolites, respectively, indicating that the effects of AKG supplementation on myoblasts were closely associated with the energy state of cells.

### 2.5. Identification of Significantly Altered Metabolic Pathways

We performed metabolic pathway analysis to identify significantly altered metabolic pathways (significant pathways) based on the levels of metabolites identified from the pairwise comparisons between the four groups of C1C12 myoblasts (Figure S7; Table 2 and Table S3). The analysis of Nor vs. Low identified 11 significant pathways: (1) Alanine, aspartate, and glutamate metabolism; (2) Glycine, serine, and threonine metabolism; (3) Glutathione metabolism; (4) d-Glutamine and d-glutamate metabolism; (5) Starch and sucrose metabolism; (6) beta-Alanine metabolism; (7) Taurine and hypotaurine metabolism; (8) Phenylalanine metabolism; (9) Phenylalanine, tyrosine and tryptophan biosynthesis; (10) Nicotinate and nicotinamide metabolism; (11) Histidine metabolism. These significant pathways were associated with energy metabolism, oxidative stress, and TCA cycle anaplerotic flux.

The analysis of Nor-A vs. Nor only identified the first five significant pathways 1–5 excluding the other six pathways 6–11. Differently, the analysis of Low-A vs. Low only identified six significant pathways: the first four pathways 1–4 shared by the comparisons of Low vs. Nor, Nor-A vs. Nor; two pathways 6–7 shared with the comparison of Low vs. Nor. Note that AKG supplementation did not significantly alter pathway 5 (Starch and sucrose metabolism) in cells cultured in a low-glucose medium, but interfered with two other pathways (beta-Alanine metabolism and taurine and hypotaurine metabolism).

**Figure 6:**
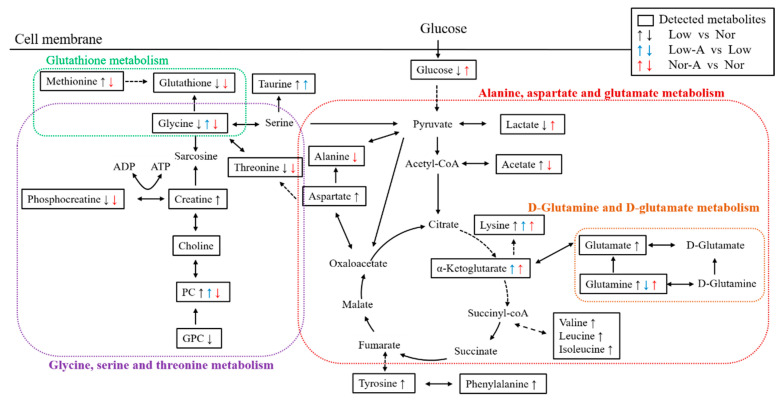
Schematic representation of significantly altered metabolic pathways identified from pairwise comparisons of Nor-A vs. Nor, Low vs. Nor, Low-A vs. Low. The up/down arrow highlights metabolites with significantly increased/decreased levels compared with the control group; dotted arrow indicates multiple biochemical reactions; solid arrow denotes a single biochemical reaction. The significantly altered metabolic pathways were identified based on the KEGG database using the MetaboAnalyst webserver.

To visualize AKG-induced changes in characteristic metabolites, we projected these metabolites onto a metabolic map based on the Kyoto Encyclopedia of Genes and Genomes (KEGG) database (Figure 6). KEGG has been extensively used as one of the main data resources to reconstruct metabolic networks and highlight significant metabolic pathways. Both the changed characteristic metabolites and significantly altered metabolic pathways provide new insights into the molecular mechanisms underlying the effects of AKG supplementation on C2C12 myoblasts.

### 2.6. Antioxidant Capacities of C2C12 Myoblasts with AKG Supplementation

**Figure 7:**
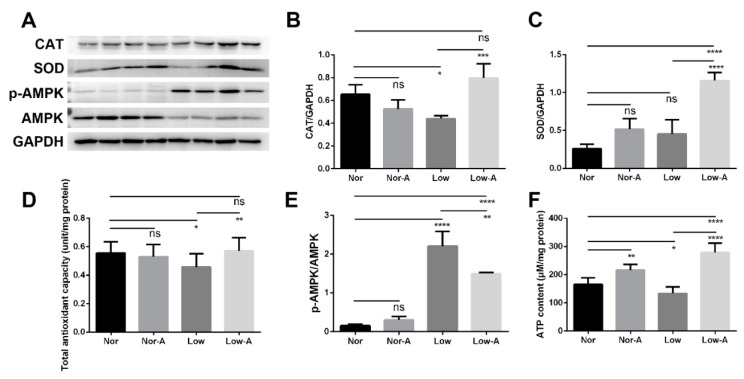
Antioxidant capacities and energy states of the four groups of C2C12 myoblasts. (**A**) Western blot analyses of antioxidant-related proteins in myoblasts. The anti-GAPDH antibody was used to standardize the amount of protein in each lane. (**B**) Expressions of the catalase (CAT) protein; (**C**) Expressions of the superoxide dismutase (SOD) protein; (**D**) Total antioxidant capacities; (**E**) Ratios of p-AMPK/AMPK; (**F**) ATP content. * *p* < 0.05, ** *p* < 0.01, *** *p* < 0.001, **** *p* < 0.0001. *n* = 4 for each group.

Oxidative stress is an important factor that greatly affects cell metabolism. To verify that AKG-enhanced proliferation and differentiation abilities of C2C12 myoblasts were correlated with AKG-alleviated cellular oxidative stress, we measured expressions of cellular superoxide dismutase (SOD) and catalase (CAT) to evaluate the myoblasts (Figure 7A–C). The SOD and CAT proteins could catalyze superoxide anions into oxygen and water, thereby alleviating the oxidative stress of cells. Low cells showed a down-regulated CAT expression and a basically identical SOD level compared to Nor cells. AKG supplementation dramatically up-regulated the expressions of SOD and CAT in myoblasts under low-glucose culture condition, but did not significantly change them under normal culture condition.

Similarly, Low cells displayed decreased total antioxidant capacity compared to Nor cells (Figure 7D). AKG supplementation distinctly increased the total antioxidant capacity of Low cells, but did not significantly change that of Nor cells. Low-A did not display a statistically different total antioxidant capacity to Nor cells, indicating that AKG supplementation restored the total antioxidant capacity of myoblasts. These results show that AKG supplementation significantly enhanced the antioxidant capacity of C2C12 myoblasts in the state of energy deficiency and, thus, alleviated cellular oxidative stress.

### 2.7. Energy States of C2C12 Myoblasts with AKG Supplementation

The ratio of p-AMPK to AMPK generally reflects the energy state of cells. Compared to Nor cells, Low cells showed a dramatically increased ratio of p-AMPK to AMPK, and a somewhat declined ATP content. In Nor cells, AKG supplementation did not obviously change the ratio of p-AMPK to AMPK, but obviously increased the ATP content (Figure 7E,F). In Low cells, AKG supplementation distinctly declined the ratio of p-AMPK to AMPK, but significantly enhanced ATP content by about one time, indicating that AKG improved the energy state of myoblasts when cellular energy was insufficient. These results demonstrate the important roles of AKG in C2C12 myoblasts in the two states of normal energy and energy deficiency.

## 3. Discussion

Myoblasts are the primary source of muscle repair and regeneration in damaged myofibers. The process of muscle regeneration involves the activation, proliferation, and differentiation of myoblasts, during which proliferation and differentiation occur simultaneously sometimes [22,23]. Concerning the mass and functions of muscle decline, supplementation of new myoblasts is a potential therapeutic strategy for repairing muscle injuries [24]. Therefore, it is vital to identify valid factors to promote the growth of myoblasts during the process of skeletal muscle regeneration, which may be beneficial to the development of ‘regenerative medicine’ for treating muscular diseases [25,26]. AKG has been recognized as a potential nutrition supplement for skeletal muscle. However, AKG-induced metabolic changes in myoblasts in different energy states remain to be systematically clarified. In the present work, we applied the conditions of normal culture and low-glucose culture to mimic the two states of normal energy and energy deficiency, respectively, and performed the cellular metabolomic analysis to exploit the effects of AKG supplementation on metabolic profiles of aqueous extracts derived from the mouse myoblast cell line C2C12. Our work reveals significant beneficial effects of AKG supplementation for promoting proliferation and differentiation of myoblasts, indicating the crucial role of AKG in enhancing the functions of skeletal muscle.

### 3.1. AKG Promotes Proliferation and Differentiation of Myoblasts

We found that AKG supplementation distinctly promoted the proliferation of myoblasts. In the two different energy states, myoblasts fused with each other to form myotubes, which became longer and thicker after AKG supplementation, indicating that AKG also promoted the myotube differentiation of myoblasts. Furthermore, under both normal culture and low-glucose culture conditions, the myoblasts exhibited enhanced expressions of MyOD1 acting as a key regulatory protein in the process of myoblast differentiation to form myotubes [27]. This result also verified the effect of AKG on promoting skeletal muscle differentiation. Even though previous studies have demonstrated that AKG can enable C2C12 myotubes to become thicker [10] or promote muscle hypertrophy [18], our study on C2C12 myoblasts provides a new perspective to mechanistically understand the beneficial effects of AKG supplements for improving functions of skeletal muscle in the two states of normal energy and energy deficiency.

### 3.2. AKG Improves Glutamate and Glutamine Metabolism

Even though AKG supplementation dramatically raised intracellular AKG levels in both Nor-A and Low-A myoblasts, this work did not focus on AKG transport since it was not a rate-limiting step involved in the AKG metabolism [28]. As a product of catabolism related to proteins and other nitrogen-containing compounds, ammonia can damage cell functions [29]. As a precursor of glutamate and glutamine, AKG can react with ammonia to form glutamate, which further reacts with ammonia to form glutamine [30]. Glutamine can serve as a carbon source to generate energy and regulate the activity of signal transduction pathways, thereby promoting cell proliferation [31,32]. As shown in Table S2, AKG supplementation significantly up-regulated the glutamine level in myoblasts under normal culture condition, although it did not significantly change the glutamate level. Additionally, myoblasts under low-glucose culture condition showed a strengthened dependence on glutamine in the lack of carbon sources, and profoundly enhanced intracellular levels of glutamate and glutamine acting as characteristic metabolites between Low and Nor cells. In fact, in the absence of glutamine breakdown, increasing the intracellular AKG level would also activate the mTORC1-related pathway to promote cellular proliferation [11]. Significantly, Low-A cells displayed a declined level of glutamine, implying that AKG supplement reduced the demand for glutamine in skeletal muscle cells in the state of energy deficiency with a high glutamine level. These results reveal that AKG supplementation significantly improves glutamate and glutamine metabolism, thereby promoting the proliferation of myoblasts cultured in both normal and low-glucose media.

### 3.3. AKG Promotes Antioxidant Capacity of Myoblasts under Low-Glucose Culture Condition

Under normal culture condition, AKG supplementation decreased cellular levels of methionine, glutathione, and glycine, implying that the glutathione metabolism was significantly down-regulated. As known, dietary supplementation with AKG can enhance the CAT and SOD expressions under oxidative stress [33]. We found that Nor-A cells did not show statistically significant differences in the CAT and SOD expressions and total antioxidant capacity from Nor cells, indicating that AKG supplementation did not significantly alter cellular oxidative stress. Compared to Nor cells, Low cells displayed a down-regulated CAT expression and a decreased total antioxidant capacity. Notably, AKG supplementation restored the total antioxidant capacity of myoblasts cultured in a low-glucose medium. Furthermore, Low-A cells displayed a significantly increased level of glycine relative to Low cells, indicating that AKG supplementation could favor the activation of cellular antioxidants in a state of energy deficiency, thereby protecting cells and maintaining cell growth and metabolism under adverse conditions. Additionally, it is known that taurine acts as an antioxidant [34,35]. In this study, Low cells exhibited a profoundly elevated intracellular level of taurine compared to Nor cells, which potentially enhanced the antioxidant ability of myoblasts under low-glucose culture condition. Significantly, AKG supplementation further raised the intracellular level of taurine, and correspondingly enhanced the antioxidant ability of cells without sufficient energy supply. It has been reported that AKG has an antioxidative function and exhibits a vital role in scavenging ROS in organisms [36]. Here, we also demonstrated that AKG supplement significantly promotes the antioxidant capacity of myoblasts under low-glucose culture condition.

### 3.4. AKG Enhances Cellular Energy Status

Acting as a source of energy, AKG can provide energy for cell processes and regulates cellular energy mechanisms. When cells were cultured in a normal medium with sufficient glucose, AKG supplementation increased intracellular levels of the upstream glucose and lactate, thereby potentially preserving the cell energy source. Compared with Nor-A cells, Nor-A cells showed a decreased level of phosphocreatine (PCr) and a basically unchanged level of creatine (Cr). PCr and Cr can be converted to each other through the reaction catalyzed by creatine kinase (CK). Note that both PCr and Cr are the two critical components of the CK/PCr system serving as the rapidly available source for ATP synthesis in skeletal muscle [37]. The decreased phosphocreatine level together with the increased ATP content in Nor-A cells, implied that AKG supplement facilitated the conversion of PCr to Cr for ATP synthase in myoblasts in the state of normal energy.

On the other hand, Low cells displayed a decreased ATP content and a remarkably decreased PCr, as well as a dramatically increased Cr level compared with Nor cells, implying that more PCr were converted to Cr to meet energy demands in cells cultured in a low-glucose medium. Significantly, AKG supplement increased the ATP content by about one time in myoblasts in the state of energy deficiency as showed in Figure 7F. Unexpectedly, Low-A cells showed a higher ATP level than Nor cells, potentially owing to both an optimal phenotype of cells achieved at a moderate glucose level and AKG supplementation [38]. Nevertheless, the underlying molecular mechanisms should be explored in detail in future.

Low-A cells exhibited reduced phosphorylation of AMPK as indicated by the distinctly declined ratio of p-AMPK to AMPK relative to Low cells, indicating that AKG supplementation enhanced antioxidant effect and improved the proliferation and metabolism of myoblasts in the state of energy deficiency. The underlying molecular mechanism also remains to be addressed in future. Under normal culture condition, AKG supplementation distinctly increased the intracellular ATP level, but did not significantly change the ratio of p-AMPK to AMPK, potentially due to the need to maintain a certain energy state in cells with sufficient energy supply. As is known, an up-regulated ATP level favors energy utilization and physiological activities of the organism such as cell proliferation and differentiation. However, it seems that several previous studies suggested apparently contradictory molecular mechanisms for further understanding the beneficial effects of AKG supplement. For example, one study suggested that AKG extends Drosophila lifespan by inhibiting mTOR and activating AMPK [39], while another study suggested that AKG activates mTOR signaling and promotes the ATP synthase in Lipopolysaccharide-challenged piglets [40]. Potentially, these differences in AKG-related mechanisms might be attributed to specific strains and genetic backgrounds relevant to the detailed experiments. Further work must be conducted to clarify the molecular mechanisms underlying the beneficial effects of AKG in different energy states.

In addition, we only performed metabolic profiling on aqueous metabolites extracted from C2C12 myoblasts. Analyzing the portion of hydrophobic metabolites in conjunction with that of aqueous metabolites would provide a much more comprehensive metabolic profile. Unfortunately, hydrophobic metabolites are usually related to poor NMR spectra with declined spectral resolution and decreased S/N ratio due to crowded, overlapped, or broadened peaks, especially peaks from lipid metabolites. It is difficult to accurately calculate integrals of most hydrophobic metabolites based on the poor NMR spectra. Further effort should be made in future to improve the NMR spectra of hydrophobic metabolites.

## 4. Materials and Methods

### 4.1. Cell Culture

Murine skeletal muscle-derived C2C12 myoblast cell line was purchased from the China Center for Typical Culture Collection (CCTCC; Wuhan, China). Cells were cultured in Dulbecco’s modified Eagle’s medium (Growth medium; GM) with glucose (normal DMEM, HyClone, Logan, UT, USA) or without glucose (no-glucose DMEM; Gibco, Gaithersburg, MD, USA) supplemented with 10% (*v*/*v*) fetal bovine serum (Gibco, Gaithersburg, MD, USA), 100 U/mL penicillin, and 100 mg/mL streptomycin. Differentiation medium (DM) was supplemented with 2% (*v*/*v*) horse serum (Gibco, Gaithersburg, MD, USA), 100 U/mL penicillin, and 100 mg/mL streptomycin. The low-glucose medium (low-glucose DMEM) was a mixture of normal DMEM and no-glucose DMEM in a ratio of 1:8. Cells were cultured in a humidified incubator containing 5% (*v*/*v*) CO_2_ at 37 °C. α-Ketoglutarate (AKG) suitable for cell culture was purchased from Sigma-Aldrich.

For obtaining C2C12 myoblasts, C2C12 cells were firstly cultured in normal DMEM to reach 50% confluence for 24 h, and then cultured in fresh normal DMEM with or without AKG supplementation (Nor-A GM, Nor GM), or in fresh low-glucose DMEM with or without AKG supplementation (Low-A GM, Low GM) for another 24 h (Figure S2A). The final concentration of AKG was 2 mm. Correspondingly, the obtained myoblasts were classified into the following four groups: Nor-A, Nor, Low-A, and Low myoblasts.

For obtaining C2C12 myotubes, C2C12 cells were firstly cultured in normal DM (the differentiation medium was replaced every two days) to form myotubes for 10 days, and then cultured in fresh normal DM with or without AKG supplementation (Nor-A DM, Nor DM), or in fresh low-glucose DM with or without AKG supplementation (Low-A DM, Low DM) for another 2 days (Figure S2B). Similarly, the obtained myotubes were also classified into the following four groups: Nor-A, Nor, Low-A, and Low myotubes.

### 4.2. MTS Cell Proliferation Assay and Morphologies of Myoblasts and Myotubes

C2C12 myoblast cells were seeded at a density of 5 × 103 cells per well in 96-well plates for 24 h by 100 μL of medium. Then, the culture medium was replaced by 100 μL of fresh medium, and the cells were incubated for another 24 h. Equivalent volumes of vehicle culture media were treated as controls. CellTiter 96 AQueous solution (MTS, Promega, Madison, WI, USA) was added to each well, and the absorbance of formazan at a wavelength of 490 nm on a microplate reader (BioTek, Winooski, VT, USA) after incubation in the dark for 3 h. In addition, C2C12 myoblasts or myotubes were washed three times using PBS to remove the dead cells. Thereafter, cell morphological images were taken randomly on a fluorescence microscope (Motic, Xiamen, China).

### 4.3. Western Blotting

C2C12 myoblast cells were lysed in a RIPA buffer (Sangon Biotech, Shanghai, China) containing protease and phosphatase inhibitor, followed by brief sonication. Cell lysates were then loaded into sodium dodecyl sulfate-polyacrylamide gel, and, thereafter, transferred onto PVDF membranes (GE, Freiburg, Germany). Membranes were blocked with 5% non-fat milk and incubated with primary antibodies overnight at 4 °C with shaking. After incubation with the secondary antibody for 1 h at room temperature, the signal was visualized by the commercially enhanced chemiluminescence reagent (ECL, Beyotime, Shanghai, China). The used antibodies were as follows: GAPDH (Proteintech, Wuhan, China), MyoD1 (Santa Cruz Biotechnology, Dallas, TX, USA), CAT (Proteintech, Wuhan, China), SOD (Proteintech, Wuhan, China), p-AMPK (CST, Boston, MA, USA), AMPK (CST, Boston, MA, USA).

### 4.4. Intracellular Metabolite Extraction and Samples Preparation

Aqueous metabolites were extracted from C2C12 myoblast cells for NMR analyses according to the protocol described previously [41]. Cells were quickly rinsed thrice by cold phosphate-buffered saline (PBS, pH 7.4) to reduce the residual medium. The residual PBS was removed immediately by vacuum suction. Subsequently, metabolic activities of cells were aborted by methanol, and cells were scraped off by a cell scraper (Costar, Washington, DC, USA). Cells were then collected into a 15 mL centrifuge tube. Thereafter, methanol, chloroform, and water in the volume ratio of 4:4:2.85 were applied in a dual-phase extraction for extracting intracellular metabolites. Only the polar phase was lyophilized and subjected to metabolomic profiling. The aqueous cell extract powder was resolved in 550 μL of phosphate buffer [50 mm, pH 7.4, 100% D_2_O, 0.05 mm sodium 3-(trimethylsilyl) propionate-2,2,3,3-*d*_4_ (TSP)], vortexed and then centrifuged at 12,000× *g* for 15 min at 4 °C. The supernatants were transferred into 5 mm NMR tubes for NMR-based metabolomic analysis.

### 4.5. NMR Measurements and Data Preprocessing

All NMR measurements were performed at 298 K on a Bruker Avance III 850 MHz NMR spectrometer (Bruker Bio Spin, Rheinstetten, Germany). One-dimensional (1D) ^1^H spectra were obtained using the pulse sequence NOESYGPPR1D [RD−G_1_−90°−t_1_−90°−τ_m_−G_2_−90°−ACQ] with water suppression during the relaxation delay (RD = 2s) and mixing time (τ_m_ = 10 ms). The short delay (t_1_) was 4 μs. Pulsed gradients G1 and G2 were used to improve water suppression quality. A spectral width of 20 ppm was used, and a total of 128 transients were collected into 64 k data points, giving an acquisition time (ACQ) of 1.93 s. Chemical shifts were referenced to the methyl group of TSP at 0 ppm. The two-dimensional (2D) ^1^H-^13^C heteronuclear single quantum coherence (HSQC) spectrum was recorded with a spectral width of 10 ppm in the ^1^H dimension and 110 ppm in the ^13^C dimension, a data matrix of 1024 × 128 points, and a relaxation delay of 1.5 s. The 2D total correlation spectroscopy (TOCSY) spectrum was recorded with a spectral width of 10 ppm in both ^1^H dimensions, a data matrix of 2048 × 256, and a relaxation delay of 1.5 s.

Phase correction, baseline correction, and resonance alignment were carried out for all 1D NMR spectra using the MestReNova 9.0 software (Mestrelab Research S.L., Santiago de Compostela, Spain). For further multivariate statistical analysis, 1D ^1^H spectral region of 9.5–0.6 ppm was segmented into bins with a width of 0.01 ppm. The water region of 4.9–4.7 ppm was excluded to eliminate distortion from the residual water resonance in all 1D spectra. Peak integrals of the segments were normalized by the sum of peak integrals to compensate for potential variations in the concentrations of samples. The sum of the peak integrals was set to 100 for each spectrum. The normalized integrals were used to represent the relative levels of assigned metabolites. For pairwise comparisons of metabolite levels between the groups, singlet or nonoverlapped resonances in each NMR spectrum were selected for computing metabolite integrals.

### 4.6. Resonance Assignments of Metabolites

Two AKG resonance regions of 2.495–2.530 ppm and 2.996–3.019 ppm were excluded to compress metabolic differences between the groups. NMR resonances of metabolites were assigned using a combination of the Chenomx NMR Suite (version 8.6, Chenomx Inc., Edmonton, AB, Canada) and Human Metabolome Database (HMDB, http://www.hmdb.ca/ accessed on 6 January 2021). In addition, 2D ^1^H-^13^C HSQC and ^1^H-^1^H TOCSY spectra were used to confirm the assigned metabolites.

### 4.7. Metabolomic Analysis

Multivariate statistical analysis was performed on 1D ^1^H-NMR spectral data of C2C12 cell extracts by using the SIMCA-P software (version 12.0.1, Umetrics, Umea, Sweden). Pareto scaling was applied to the normalized NMR spectral data to enhance the significances of low-level metabolites without noise enlargement. Then, principal component analysis (PCA) was conducted to examine grouping trends and reveal metabolic differences. Moreover, orthogonal partial least-squares discriminant analysis (OPLS-DA) was conducted to check grouping trends and improve group separation. The cross-validation was performed to measure the robustness of the OPLS-DA model with a response permutation test (200 times). The reliability of the OPLS-DA model was raised as the R2 and Q2 approached 1. Important metabolites were identified with VIP > 1 from the OPLS-DA model.

Univariate data analysis was conducted on the relative levels of the assigned metabolites between the groups, which were calculated based on the integrals of the metabolites relative to the sum of metabolite integrals. We quantitatively compared the relative levels of the metabolites between the groups using a two-tailed Student’s *t*-test with the Graphpad Prism software (version 6.0, La Jolla, San Diego, CA, USA). Data were expressed as the mean ± SD. Metabolites with *p* < 0.05 were identified to be differential metabolites. Characteristic metabolites were determined by a combination of the differential metabolites and important metabolites described above.

The metabolic pathway analysis was performed on the MetaboAnalyst 5.0 webserver (https://www.metaboanalyst.ca accessed on 6 January 2021), using a combination of metabolite sets enrichment analysis (*p* < 0.05) and pathway topological analysis (pathway impact value > 0.2). Significantly altered metabolic pathways were identified for pairwise comparisons of Nor-A vs. Nor, Low vs. Nor, Low-A vs. Low based on the relative levels of the assigned metabolites.

### 4.8. Measurement of Cellular Total Antioxidant Capacity (T-AOC)

Cells were centrifuged at 3000 g and the supernatant was discarded after digestion. Then, 1.2 mL of lysis buffer was added to the pellet to lyse the cells. Lysates were centrifuged for 10 min at 12,000× *g*. Cellular T-AOC was assayed by the total antioxidant capacity kit (Nanjin Jiancheng Bioengineering Institute, Nanjing, China). BCA protein assay (Beyotime, Shanghai, China) was used to measure the amount of T-AOC per mg protein.

### 4.9. Measurement of Intracellular ATP Content

After 2.4 mL of lysis buffer was added to each dish of cells, lysates were collected into an Eppendorf tube. Eppendorf tubes were spun in the centrifuge for 5 min at 12,000× *g*. The ATP content was measured by the luciferase method using the ATP assay kit according to the manufacturer’s instruction. The protein concentration in each sample was detected by the BCA protein assay (Beyotime, Shanghai, China) to calculate the ATP concentration per mg protein.

## 5. Conclusions

In summary, we have demonstrated AKG-induced metabolic changes of skeletal muscle cells in the two different energy states. AKG supplementation significantly alters cellular metabolisms in different ways, and enhances the proliferation and differentiation of C2C12 myoblasts through glutamine metabolism, oxidative stress, and energy metabolism in both normal energy and energy deficiency states. Under the condition of sufficient energy supply, AKG supplementation up-regulates the intracellular glutamine level, enhances the cellular energy status, and maintains the antioxidant capacity of myoblasts. Under the circumstance of energy deficiency, AKG serves as a metabolic substrate to reduce the glutamine dependence of cells, greatly enhances the antioxidant capacity of myoblasts, and significantly elevates the intracellular ATP level, thereby ensuring the normal growth and metabolism of cells without sufficient energy supply. Our results shed light on the molecular mechanisms underlying the beneficial effects of AKG on skeletal muscle cells. Our work may be helpful to the development of AKG applications in clinical treatment and dietary supplementation.

## Figures and Tables

**Table 1:** Characteristic metabolites between the four groups of C2C12 myoblasts.

Metabolites	Groups		
	Low vs. Nor	Low-A vs. Low	Nor-A vs. Nor
Glutathione	ns	ns	**↓**
Glutamate	**↑**	ns	ns
AKG	ns	**↑**	**↑**
Pyroglutamate	ns	ns	**↑**
Glutamine	**↑**	**↓**	ns
Aspartate	**↑**	ns	ns
Creatine	**↑**	ns	ns
Phosphocreatine	**↓**	ns	**↓**
PC	**↑**	**↑**	**↓**
GPC	**↓**	ns	ns
Taurine	**↑**	**↑**	ns
Myoinositol	ns	**↓**	**↓**
Glucose	ns	ns	**↑**
Glycine	**↓**	**↑**	**↓**
Lactate	**↓**	ns	**↑**
Threonine	ns	ns	**↓**

Characteristic metabolites were identified with two criteria of *p* < 0.05 and VIP > 1 calculated from the OPLS-DA models. Red/Blue arrows demote increased/decreased levels of metabolites.

**Table 2:** Significantly altered metabolic pathways identified from the pairwise comparisons between the Nor-A and Nor groups, the Low and Nor groups, the Low-A and Low groups.

NO	Metabolic Pathway	Low vs. Nor	Low-A vs. Low	Nor-A vs. Nor
1	Alanine, aspartate and glutamate metabolism	√	√	√
2	Glycine, serine, and threonine metabolism	√	√	√
3	Glutathione metabolism	√	√	√
4	d-Glutamine and d-glutamate metabolism	√	√	√
5	Starch and sucrose metabolism	√		√
6	beta-Alanine metabolism	√	√	
7	Taurine and hypotaurine metabolism	√	√	
8	Phenylalanine metabolism	√		
9	Phenylalanine, tyrosine, and tryptophan biosynthesis	√		
10	Nicotinate and nicotinamide metabolism	√		
11	Histidine metabolism	√		

## Data Availability

Data is contained within the article or supplementary material.

## Supplementary Materials

The following data are available online, Figure S1: Relative cell viabilities of C2C12 myoblasts cultured in normal medium with different concentrations of AKG; Figure S2: Schematic representation of the experimental design; Figure S3: Morphologies of C2C12 myotubes cultured in normal DM and low-glucose DM with or without AKG supplementation; Figure S4: Representative 2D ^1^H-^13^C HSQC spectrum of aqueous extracts derived from C2C12 myoblasts recorded on 850 MHz NMR spectrometer; Figure S5: Representative 2D ^1^H-^1^H TOCSY spectrum of aqueous extracts derived from C2C12 myoblasts recorded on 850 MHz NMR spectrometer; Figure S6: Cross-validation plots of OPLS-DA models of Low vs. Nor, Low-A vs. Low, Nor-A vs. Nor; Figure S7: Significantly altered metabolic pathways of Low vs. Nor, Low-A vs. Low, Nor-A vs. Nor; Table S1: Resonance assignments of aqueous extracts derived from C2C12 myoblasts; Table S2: Comparisons of metabolite levels between the Nor, Nor-A, Low and Low-A groups of C2C12 myoblasts based on relative NMR integrals with Student’s *t*-test analysis.

## Sample Availability

Samples of the NMR experiments are available from the authors.
