# Supplementary material for: NMR-Based Metabolomic Analysis for the Effects of α-Ketoglutarate Supplementation on C2C12 Myoblasts in Different Energy States
Source: Molecules. 2021 Mar 25;26(7):1841. doi: 10.3390/molecules26071841 (PMC8037044; doi:10.3390/molecules26071841)
Supplement: Supplementary file 1 [file molecules-26-01841-s001.pdf]

Supplementary Materials

Figure S1.

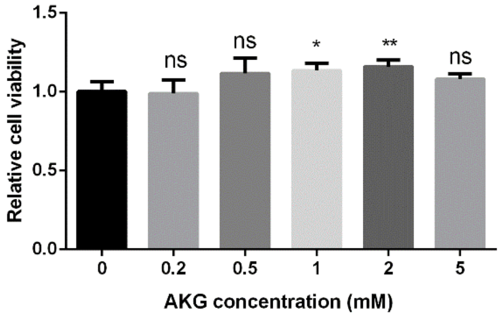

Figure S1. Relative cell viabilities of C2C12 myoblasts cultured in normal growth medium with different concentrations of AKG. $n = 5$ for each group. $^{*}p < 0.05$, $^{**}p < 0.01$.

Figure S2.

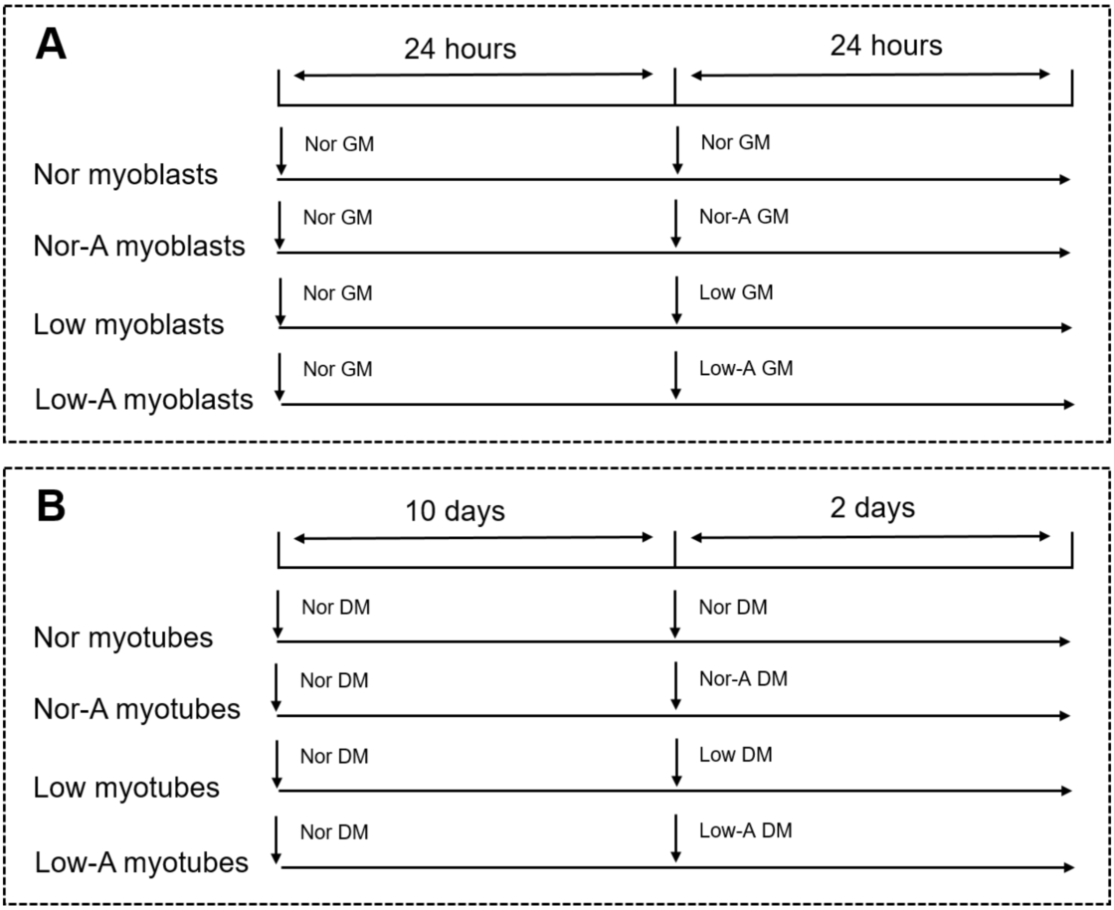

Figure S2. Schematic representation of the experimental design. (A) C2C12 cells were firstly cultured in normal growth medium (Nor GM) to reach 50% confluence for 24 hours and then cultured in four different media for another 24 hours. Nor-A GM: normal growth medium supplemented with AKG; Low GM: low-glucose growth medium; Low GM: low-glucose growth medium supplemented with AKG. **(B)** C2C12 cells were firstly cultured in normal differentiation medium (Nor DM) to form myotubes for 10 days and then cultured in four different media for another 2 days. Nor-A GM: normal growth medium supplemented with AKG; Low GM: low-glucose growth medium; Low GM: low-glucose growth medium supplemented with AKG.

Figure S3.

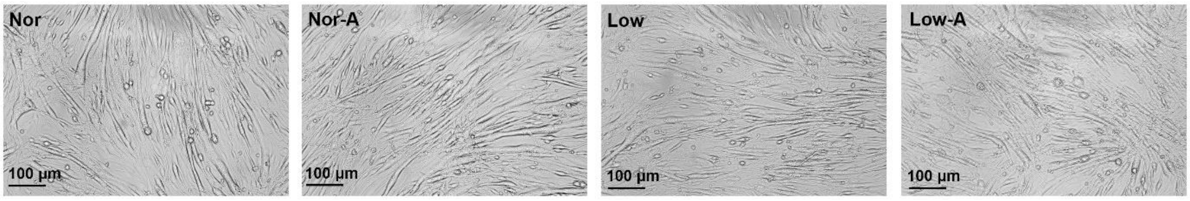

Figure S3. Morphologies of C2C12 myotubes cultured in normal DM and low-glucose DM with or without AKG supplementation.

Figure S4.

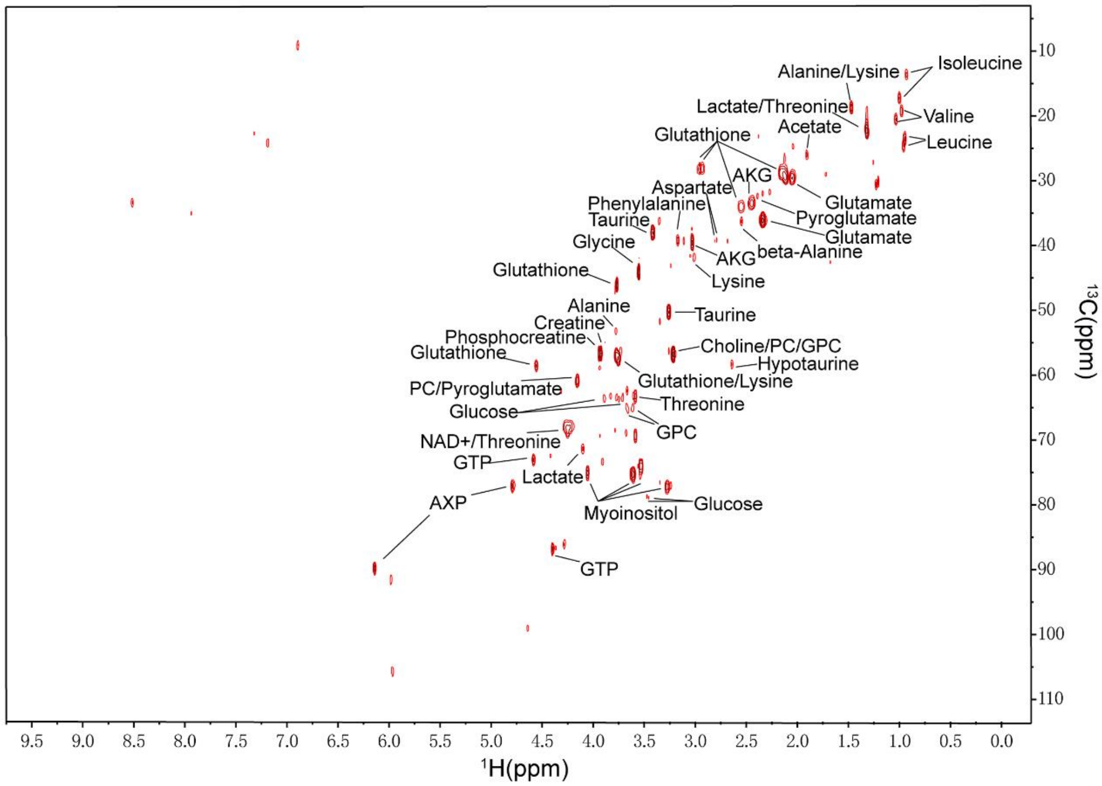

Figure S4. Representative 2D ^{1}H-^{13}C HSQC spectrum of aqueous extracts derived from C2C12 myoblasts recorded on 850 MHz NMR spectrometer.

Figure S5

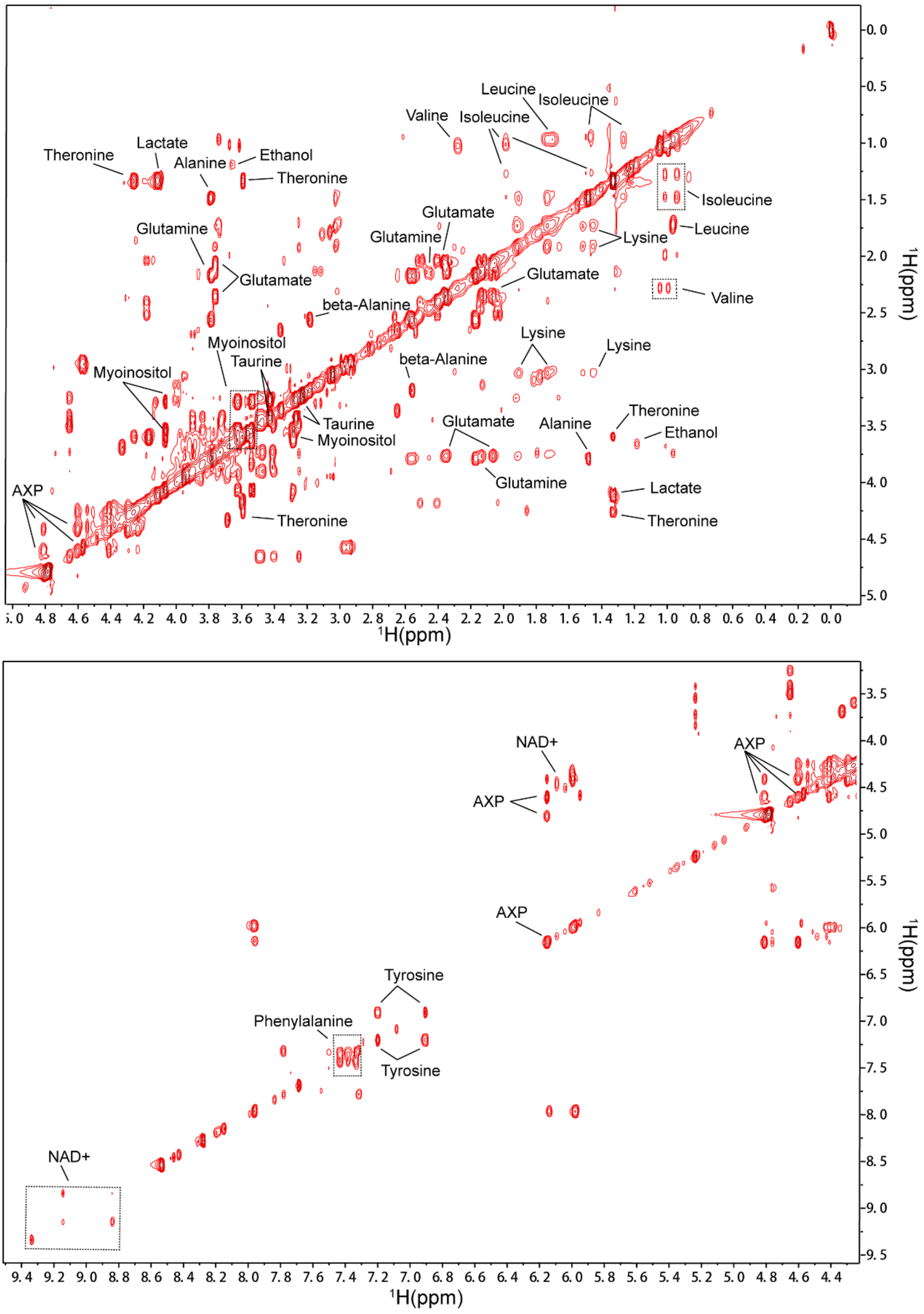

Figure S5. Representative 2D ^{1}H-^{1}H TOCSY spectrum of aqueous extracts derived from C2C12 myoblasts recorded on 850 MHz NMR spectrometer.

Figure S6

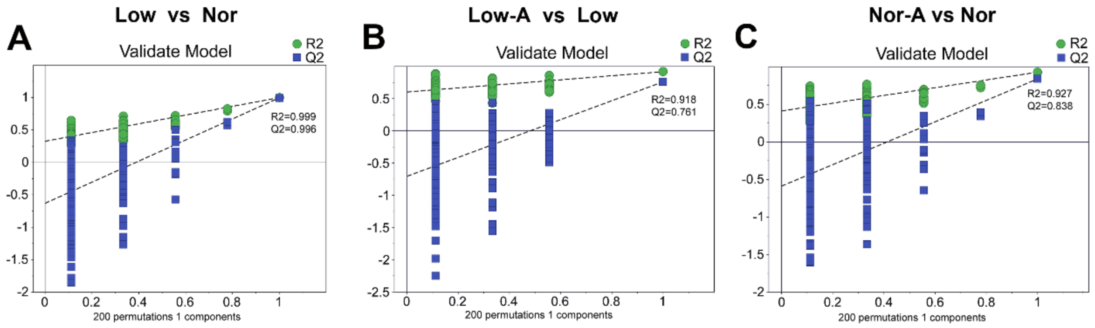

Figure S6. Cross-validation plots of OPLS-DA models of Low vs Nor, Low-A vs Low, Nor-A vs Nor.

Figure S7

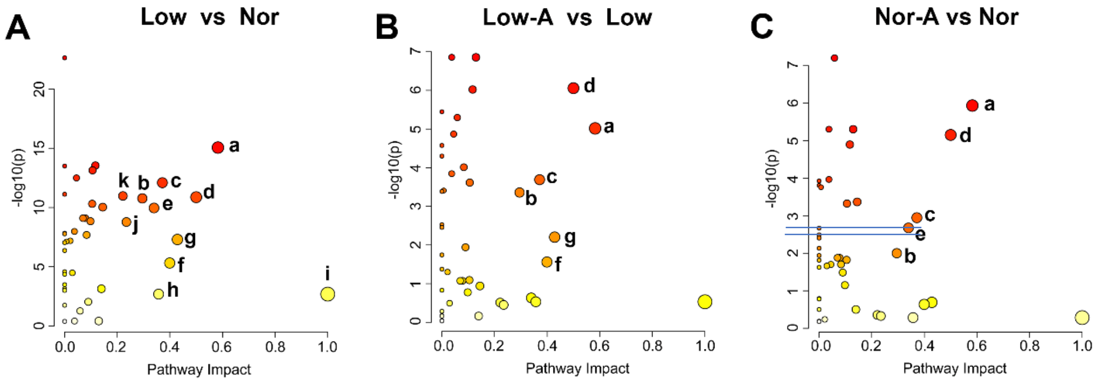

Figure S7. Significantly altered metabolic pathways of Low vs Nor, Low-A vs Low, Nor-A vs Nor. Characters in the panels represent significantly altered metabolic pathways: a. Alanine, aspartate and glutamate metabolism; b. Glycine, serine and threonine metabolism; c. Glutathione metabolism; d. D-Glutamine and D-glutamate metabolism; e. Starch and sucrose metabolism; f. beta-Alanine metabolism; g. Taurine and hypotaurine metabolism; h. Phenylalanine metabolism; i. Phenylalanine, tyrosine and tryptophan biosynthesis; j. Nicotinate and nicotinamide metabolism; k. Histidine metabolism.

Table S1. Resonance assignments of aqueous extracts derived from C2C12 myoblasts.

NO.	Metabolites	δ ^{1}H (ppm) and multiplicity	Moieties
1	Leucine	0.96 (d), 0.97 (d),1.69 (m), 1.70 (m), 1.73 (m), 3.73 (m)	α-CH_3, α-CH_3, γ-CH, β-CH_2, α-CH
2	Isoleucine	0.94 (t), 1.01(d), 1.21 (m), 1.42 (m), 2.00 (m), 3.67 (d)	δ-CH_3, γ-CH_3, half γ-CH_2, half γ-C H_2, β-CH, α-CH
3	Valine	0.99 (d), 1.05 (d), 2.26 (m), 3.60 (d)	γ-CH_3, γ-CH_3, β-CH, α-CH
4	Ethanol	1.17 (t), 3.65 (q)	δ-CH_3, CH_2
5	Threonine	1.30 (d), 3.58 (d), 4.24 (m)	γ-CH_2, β-CH
6	Lactate	1.33 (d), 4.11 (q)	β-CH_3, α-CH
7	Alanine	1.47 (d), 3.78 (q)	β-CH_3, α-CH
8	Acetate	1.91 (s)	CH_3
9	Methionine	1.98 (m), 2.13 (s), 2.17 (m), 2.66 (dd), 3.78 (m)	δ-CH_3, γ-CH_2, β-CH_2
10	Pyroglutamate	2.05 (m),2.39 (d),2.51 (m), 4.18 (dd)	β-CH, γ-CH_2, β-CH, α-CH
11	Glutamate	2.08 (m), 2.12 (m), 2.34 (m), 2.37 (m), 3.75 (m)	Half β-CH_2, half β-CH_2, half γ-CH_2, half γ-CH_2, α-CH
12	Lysine	1.43 (m),1.49 (m),1.70 (m), 1.91 (m), 3.02 (t), 3.75 (t)	half γ-CH_2, half γ-CH_2, δ-CH_2, β-CH_2, ε-CH_2, α-CH
13	Glutamine	2.13 (m), 2.45 (m), 3.77 (t)	γ-CH_2, β-CH_2, α-CH
14	Glutathione	2.15 (m), 2.55 (m), 2.96 (m), 3.77 (m), 4.56 (m)	β-CH_2, γ-CH_2, CH_2-SH, α-CH &CH_2–NH, CH–NH
15	α-Ketoglutarate (AKG)	2.45 (t), 3.01 (t)	CH_2, CH_2
16	beta-Alanine	2.54 (t), 3.17 (t)	CH_2, CH_2
17	Aspartate	2.68 (dd); 2.81 (dd); 3.90 (dd)	β-CH_2; α-CH
18	Creatine	3.04 (s), 3.93 (s)	N-CH_3, CH_2
19	Phosphocreatine	3.05 (s), 4.05 (s)	N–CH_3, CH_2
20	Tyrosine	3.05 (dd), 3.19 (dd), 6.92 (d), 7.19 (d)	half β-CH_2, half β-CH_2, β-CH, α-CH
21	Phenylalanine	3.12 (dd), 3.30 (dd), 3.99 (dd), 7.33 (d), 7.37 (t), 7.43 (t)	α-CH, half β-CH_2, half β-CH_2, α-CH, β-CH, γ-CH
22	Choline	3.21 (s), 3.51 (dd), 4.04 (t)	N-$(CH_3)_3$, α-CH_2, CH2OH
23	Phosphocholine （PC)	3.22 (s), 3.60 (t), 4.18 (m）	N–(CH3)$_3$, N–CH_2, CH_2OH
24	sn-Glycero-3-phosphocholine (GPC）	3.23 (s), 3.60 (dd), 3.68 (dd), 3.87 (m), 3.94 (m), 4.33 (m)	N–(CH3)$_3$, half $^1CH_2$, $^2CH_2$, half $^2CH_2$, half $^3CH_2$, half $^3CH_2$, $^1CH_2$
25	Taurine	3.24 (t), 3.41 (t)	$^1CH_2$, $^2CH_2$
26	Myo-inositol	3.28 (t),3.53 (dd),3.63 (t),4.07 (t)	^{2}CH,^{4,6}CH,^{1,3}CH,^{5}CH
27	Glucose	β((3.24 (dd), 3.48 (t), 3.90 (dd)), α(3.54 (dd), 3.71 (t), 3.72 (dd), 3.83 (m))	β (H_2, H_3, H_5), α(H_2, H_3, H_6)

28	Glycine	3.57 (s)	α-CH_2
29	UDP-glucose	5.62 (dd), 6.0 (m）	CH, 2CH
30	GTP	5.92 (d), 8.1 (s)	CH, CH
31	NAD+	6.03 (d), 6.08 (s), 8.16 (s), 8.20 (m), 8.41 (s), 8.82 (d), 9.13 (d), 9.32 (s)	NH_2, NH_2(CO), d-CH, β-CH, ^{2}CH, γ-CH, α-CH
32	AXP	6.14 (d), 8.27 (s), 8.58 (s)	NH_2, δ-CH, 2CH
33	Histidine	7.06 (s),7.85 (s)	^{5}CH,^{2}CH
34	Formate	8.46 (s)	CH

[a] Multiplicity: s, singlet; d, double; t, triplet; q, quartet; m, multiple; dd, double of double.

Table S2. Comparisons of metabolite levels between the Nor, Nor-A, Low and Low-A groups of C2C12 myoblasts based on relative NMR integrals with Student's t-test analyse.

Metabolites	Mean ± Standard error			
	Nor	Nor-A	Low	Low-A
Leucine	1.235 ± 0.0172	1.218 ± 0.009	1.391 ± 0.038 **	1.393 ± 0.023
Isoleucine	0.495 ± 0.013	0.492 ± 0.007	0.591 ± 0.020 **	0.586 ± 0.010
Valine	0.562 ± 0.013	0.560 ± 0.007	0.653 ± 0.018 ***	0.648 ± 0.009
Ethanol	0.092 ± 0.012	0.077 ± 0.008	0.107 ± 0.007	0.145 ± 0.012 #
Alanine	2.199 ± 0.090	1.919 ± 0.038 *	2.280 ± 0.032	2.257 ± 0.042
Acetate	0.243 ± 0.006	0.222 ± 0.006 *	0.343 ± 0.011 ****	0.320 ± 0.020
Glutathione	0.770 ± 0.013	0.686 ± 0.018 **	0.722 ± 0.008 **	0.705 ± 0.007
Glutamate	2.528 ± 0.068	2.453 ± 0.095	6.558 ± 0.028 ****	6.663 ± 0.068
AKG	0.151 ± 0.010	0.363 ± 0.020 ****	0.123 ± 0.009	0.242 ± 0.015 ####
Pyroglutamate	0.629 ± 0.017	0.789 ± 0.021 ****	0.312 ± 0.018 ****	0.303 ± 0.013
Glutamine	0.121 ± 0.009	0.176 ± 0.008 ***	1.653 ± 0.017 ****	1.528 ± 0.022 ###
Methionine	0.529 ± 0.007	0.500 ± 0.008 *	0.667 ± 0.005 ****	0.680 ± 0.005
Aspartate	0.115 ± 0.006	0.118 ± 0.005	0.574 ± 0.006 ****	0.565 ± 0.014
Lysine	0.238 ± 0.007	0.264 ± 0.006 *	0.310 ± 0.011 ****	0.279 ± 0.009 #
Creatine	1.181 ± 0.118	1.105 ± 0.090	1.788 ± 0.035 ***	1.799 ± 0.033
Phosphocreatine	1.917 ± 0.065	1.663 ± 0.050 **	1.155 ± 0.035 ****	1.261 ± 0.041 (0.0673)
beta-Alanine	0.578 ± 0.007	0.554 ± 0.015	0.553 ± 0.006 *	0.585 ± 0.007 ##
Choline	0.171 ± 0.016	0.162 ± 0.005	0.156 ± 0.009	0.131 ± 0.008
PC	3.075 ± 0.077	2.633 ± 0.063 ***	4.029 ± 0.032 ****	4.419 ± 0.064 ####
GPC	1.218 ± 0.031	1.197 ± 0.036	0.622 ± 0.014 ****	0.597 ± 0.008
Taurine	3.442 ± 0.055	3.357 ± 0.034	3.997 ± 0.019 ****	4.092 ± 0.023 ##
Myoinositol	1.438 ± 0.026	1.210 ± 0.022 ****	1.414 ± 0.006	1.314 ± 0.010 ####
Glucose	0.204 ± 0.018	0.297 ± 0.025 **	0.018 ± 0.001 ****	0.018 ± 0.002
Glycine	3.460 ± 0.043	3.217 ± 0.058 **	2.914 ± 0.018 ****	3.086 ± 0.015 ####
Lactate	1.664 ± 0.056	2.067 ± 0.085 **	0.239 ± 0.020 ****	0.191 ± 0.015
Threonine	1.391 ± 0.049	1.215 ± 0.028 **	1.081 ± 0.04 ***	1.165 ± 0.027
GTP	0.104 ± 0.003	0.095 ± 0.002 *	0.094 ± 0.001 **	0.101 ± 0.002 #
UDP-Glucose	0.033 ± 0.002	0.027 ± 0.002 *	0.010 ± 0.001 ****	0.007 ± 0.001
Tyrosine	0.151 ± 0.003	0.148 ± 0.001	0.168 ± 0.003 ***	0.170 ± 0.002
Phenylalanine	0.253 ± 0.007	0.256 ± 0.002	0.279 ± 0.006 *	0.292 ± 0.006
Histidine	0.020 ± 0.001	0.022 ± 0.001	0.025 ± 0.001 **	0.023 ± 0.000
NAD+	0.038 ± 0.002	0.036 ± 0.002	0.046 ± 0.001 ***	0.048 ± 0.001
Formate	0.036 ± 0.002	0.035 ± 0.002	0.046 ± 0.003 **	0.043 ± 0.002
AXP	0.647 ± 0.013	0.584 ± 0.014 **	0.753 ± 0.004 ****	0.756 ± 0.010

[a] Relative levels of the metabolites were quantified based on the data normalized on the total area sum.

[b] p values were calculated from independent samples t-test, statistical significances: * $p < 0.05$, ** $p < 0.01$, *** $p < 0.001$, **** $p < 0.0001$ compared to the Nor group, # $p < 0.05$, ## $p < 0.01$, #### $p < 0.0001$ compared to the Low group. Red/blue colors denote increased/decreased metabolites, respectively. $n = 9$ for each group.

Table S3. Relevant information of significantly altered metabolic pathways and the most influential metabolites.

NO.	Metabolic Pathway	Pathway impact	Match Status	Matched metabolites	p value		
					Low vs Nor	Low-A vs Low	Nor-A vs Nor
1	Alanine, aspartate and glutamate metabolism	0.583	5/28	Alanine, Aspartate, Glutamate, AKG, Glutamine,	8.664E-16	9.637E-6	1.157E-6
2	Glycine, serine and threonine metabolism	0.295	4/34	Choline, Threonine, Glycine, Creatine,	1.689E-11	4.382E-4	9.990E-3
3	Glutathione metabolism	0.371	4/28	Glycine, Glutathione, Glutamate, 5-Oxoproline	8.026E-13	2.044E-4	1.130E-3
4	D-Glutamine and D-glutamate metabolism	0.500	3/6	Glutamine, Glutamate, AKG	1.339E-11	8.777E-7	7.013E-6
5	Starch and sucrose metabolism	0.014	1/15	UDP-glucose	7.944E-10	8.445E-2	2.138E-3
6	beta-Alanine metabolism	0.399	3/21	Aspartate, beta-Alanine, Histidine	4.828E-6	2.754E-2	2.315E-1
7	Taurine and hypotaurine metabolism	0.429	1/8	Taurine	5.011E-8	6.268E-3	2.040E-1
8	Phenylalanine metabolism	0.357	2/12	Phenylalanine, Tyrosine	2.044E-3	2.943E-1	5.206E-1
9	Phenylalanine, tyrosine and tryptophan biosynthesis	1.000	2/4	Phenylalanine, Tyrosine	2.0443E-3	2.943E-1	5.206E-1
10	Nicotinate and nicotinamide metabolism	0.235	2/15	Aspartate, NAD+	1.669E-9	3.553E-1	4.713E-1
11	Histidine metabolism	0.221	3/16	Histidine, Aspartate, Glutamate	1.056E-11	3.079E-1	4.414E-1

[a] The metabolic pathway analysis was performed on the MetaboAnalyst 5.0 webserver (https://www.metaboanalyst.ca), using a combination of metabolite sets enrichment analysis ($p < 0.05$) and pathway topological analysis (pathway impact value > 0.2).
